# Modulation of Craving Related Brain Responses Using Real-Time fMRI in Patients with Alcohol Use Disorder

**DOI:** 10.1371/journal.pone.0133034

**Published:** 2015-07-23

**Authors:** Susanne Karch, Daniel Keeser, Sebastian Hümmer, Marco Paolini, Valerie Kirsch, Temmuz Karali, Michael Kupka, Boris-Stephan Rauchmann, Agnieszka Chrobok, Janusch Blautzik, Gabi Koller, Birgit Ertl-Wagner, Oliver Pogarell

**Affiliations:** 1 Department of Psychiatry and Psychotherapy, Ludwig-Maximilians-University Munich, Munich, Germany; 2 Institute for Clinical Radiology, Ludwig-Maximilians-University Munich, Munich, Germany; 3 Department of Neurology, Ludwig-Maximilians-University Munich, Munich, Germany; University of Ariel, ISRAEL

## Abstract

**Literature:**

One prominent symptom in addiction disorders is the strong desire to consume a particular substance or to display a certain behaviour (craving). Especially the strong association between craving and the probability of relapse emphasises the importance of craving in the therapeutic process. Neuroimaging studies have shown that craving is associated with increased responses, predominantly in fronto-striatal areas.

**Aim and Methods:**

The aim of the present study is the modification of craving-related neuronal responses in patients with alcohol addiction using fMRI real-time neurofeedback. For that purpose, patients with alcohol use disorder and healthy controls participated once in neurofeedback training; during the sessions neuronal activity within an individualized cortical region of interest (ROI) (anterior cingulate cortex, insula, dorsolateral prefrontal cortex) was evaluated. In addition, variations regarding the connectivity between brain regions were assessed in the resting state.

**Results and Discussion:**

The results showed a significant reduction of neuronal activity in patients at the end of the training compared to the beginning, especially in the anterior cingulate cortex, the insula, the inferior temporal gyrus and the medial frontal gyrus. Furthermore, the results show that patients were able to regulate their neuronal activities in the ROI, whereas healthy subjects achieved no significant reduction. However, there was a wide variability regarding the effects of the training within the group of patients. After the neurofeedback-sessions, individual craving was slightly reduced compared to baseline. The results demonstrate that it seems feasible for patients with alcohol dependency to reduce their neuronal activity using rtfMRI neurofeedback. In addition, there is some evidence that craving can be influenced with the help of this technique.

**Future Prospects:**

In future, real-time fMRI might be a complementary neurophysiological-based strategy for the psychotherapy of patients with psychiatric or psychosomatic diseases. For that purpose, the stability of this effect and the generalizability needs to be assessed.

## Literature

Alcohol dependence is characterised by criteria such as tolerance development, withdrawal symptoms, drug craving and reduced control of alcohol intake [1]. One of the most prominent symptoms in addiction disorders is the strong desire to consume a particular substance (craving). Craving ranks among the most important aspects of relapse. The exposure to stimuli, which have regularly been associated with drug consumption as well as addiction behaviour, can become conditioned cues eliciting conditioned responses, such as drug consumption and craving [2,3,4]. The strong association between craving and the risk of relapse emphasises the importance of craving within the therapeutic process.

Neuroimaging studies have revealed some evidence for the association of ACC and medial frontal areas during cue exposure [5]. In addition, the orbitofrontal cortex [OFC] [6,7], the dorsolateral prefrontal cortex [DLPFC] [8], the thalamus [8] and the striatum [5,9] seem to be affected. Grusser and colleagues (2004) have described that a neuronal network seems to be activated by drug-associated and alcohol-associated stimuli, including the ACC and the adjacent medial prefrontal cortex, the ventral/dorsal striatum, the amygdala and the hypothalamus. In addition, increased BOLD signal responses in the striatum and the medial PFC were related to the subsequent relapse rate [10].

Li and colleagues (2013) have demonstrated that the reduction of neuronal activity in addiction-associated brain areas can be accompanied by reduced craving [11]. For that purpose, neurofeedback with functional Magnetic Resonance Imaging (fMRI) was used. In summary, the concept of neurofeedback suggests possibilities to voluntarily influence brain activity. The effect of neurofeedback could well be explained by the basis of behavioural therapy (operant conditioning). Basically, it is a learning process which leads to the strengthening of distinct behaviours [12]. Up to now, predominantly electroencephalography was used for neurofeedback, e.g. in patients with attention deficit/hyperactivity disorder [13,14,15,16,17], for the communication with severely paralysed patients [18,19,20,21], or to suppress epileptic activity [22]. However, with EEG-neurofeedback it is difficult to modulate the activity in small brain areas because of the low spatial resolution. In addition, the activity of subcortical regions cannot be modulated.

Real-time fMRI (rtfMRI) provides a relatively new approach to measure neuronal responses and neurofeedback (NF) using functional Magnetic Resonance Imaging (fMRI). Using real-time fMRI (rtfMRI) the level of neuronal activity in circumscribed brain regions can be fed back to participants [23,24,25]. Thus, the goal is not just activation, but an enhancement of control over brain activation corresponding with an enhancement of control over the related cognitive process [26]. Several studies have demonstrated that the successful manipulation of activity in the sensory-motor area is suitable to increase the BOLD signal in motor, somatosensory and supplementary motor areas [27,28,29,30]. The main focus of more recent studies has been the manipulation of blood oxygenation level dependent (BOLD) responses in areas which are related to cognitive and emotional processes [25,31,32,33,34,35,36,37,38].

It has been shown that rtfMRI-associated neuronal variations can lead to cognitive and emotional changes [39]. Several studies using control conditions indicated that unspecific neurofeedback-training does not lead to learned regulation of localised brain activity: persons who received rtfMRI information derived from brain regions that either are supposed to be not involved in the processing of the present task [39,40,41] or from a previously tested participant [40] were not able to control their brain responses accordingly [39,40];their behaviour did not change. In a recent study, real-time fMRI was used in subjects with contamination anxiety [42]. The results indicated reduced activity in brain areas which have been associated with emotional processing (e.g. insula and adjacent areas, hippocampus, amygdala, substantia nigra, thalamus) after neurofeedback training. By contrast, BOLD responses in brain areas have been linked to emotion-regulation and cognitive control (e.g. prefrontal cortex). These variations could not be shown in the group which received neurofeedback of a brain region that is not associated with the task (sham neurofeedback). In addition, a modulation of functional connectivity in anxiety-related brain areas was demonstrated [42].

Until now, there have only been a few studies focusing on the effect of rtfMRI in patients e.g. patients with chronic pain and major depression [40,43, 44]. Li and colleagues (2013) examined the ability of patients with tobacco use disorder to regulate the activity in frontal brain areas (e.g. ACC, middle prefrontal cortex). Smokers were able to reduce the activity in the ACC during the presentation of addiction-related cues. The reduction of neuronal activity was accompanied by reduced craving [11]. Hence, at least a temporary reduction of craving was demonstrated.

Canterberry and colleagues (2013) examined neurofeedback as a tool to facilitate self-regulation of craving in nicotine-dependent cigarette smokers. Altogether, neurofeedback led to decreases in self-reported craving and activation in the ACC. Dependence severity predicted response to neurofeedback at the last visit: individuals with lower nicotine-dependence severity were more successful in reducing ACC activation [45]. In addition, Hanlon and colleagues (2013) demonstrated that treatment-seeking smokers are more effective at decreasing activity in functionally defined regions involved in *craving* (e.g. ventral anterior cingulate cortex rather than increasing activity in regions involved in “resisting” (e.g. dorsal medial prefrontal cortex) [46].

Ruiz and colleagues (2013) examined rtfMRI-responses in patients suffering from schizophrenia. Their results demonstrated abnormal connectivity between brain regions e.g. fronto-temporal areas or variations in the connectivity, especially among frontal and limbic brain areas. The results of a resting state paradigm indicated that self-regulation capability was negatively correlated with the severity of negative symptoms and the duration of the illness. In addition, self-regulation led to changes in emotion detection. At the end of the training, effective connections between insula cortex, amygdala and medial PFC were enhanced [47].

The aim of the present project was to investigate the feasibility of rtfMRI neurofeedback to influence both neuronal responses and craving to addiction-related cues in patients with alcohol use disorder. For that purpose, brain responses in areas, which are associated with craving were modulated in patients with alcohol dependence. The main hypotheses were that BOLD responses in circumscribed brain areas can be modulated with the aid of rtfMRI neurofeedback and that these processes are related to decreased subjective craving ratings after neurofeedback sessions. In addition, we speculated that functional differences regarding true rtfMRI could be enhanced compared to baseline in the sense that an improved down-regulation of hyper-connectivity in brain regions like the insular cortex, ACC or DLPFC may be of clinical relevance (Seo et al. 2013). To further specify this hypothesis we defined seed-based regions of interest (ROIs) partly belonging to the Default Mode Network (DMN) like the ACC and craving-relevant regions beyond the DMN like the insular cortex. We further hypothesise that a possible change in the BOLD signal of these ROIs may change seed-based functional connectivity in broader parts of the human brain.

## Methods

### Subjects

#### Characteristics of the true neurofeedback group

The study included the investigation of 13 patients with alcohol addiction and 14 healthy controls. All patients were recruited from a specialised therapeutic programme for alcohol-addiction: stationary detoxification in a ward specialising in drug addiction at the Department of Psychiatry and Psychotherapy; LMU Munich. All patients were diagnosed by two experienced and licensed psychiatrists in line with the criteria of the international classification of disease (ICD 10), F10.2 requiring the presence of at least three of six criteria. Participation in the study did not influence treatment strategies for patients; the participation in the neurofeedback-study was an add-on to the standard treatment.

The study was approved by the ethics committee of Medical Department of the Ludwig-Maximilians-University Munich. The investigation was carried out in accordance with the Declaration of Helsinki. Written informed consent was obtained from each participant after procedures had been fully explained. The consent procedures were approved by the ethics committee.

The key exclusion criteria for MRI were respected (e.g. claustrophobia, pregnancy, metal, cardiac pacemaker). The inclusion criteria for both groups were age between 18–60 years. In healthy controls an additional exclusion criterion was a lifetime diagnosis of a neurological and / or psychiatric disorder. In addition, patients with the ICD-10 diagnosis of alcohol addiction were included (F 10.2). Patients with a further neurological and / or psychiatric disorder were not included in the study.

#### Characteristics of the sham neurofeedback group

Apart from these two patients and 5 controls were included in a control condition with *sham* neurofeedback. The patients of the *sham* neurofeedback rtfMRI were 43 years (men) and 54 years (woman) old. In the sham control group 4 men and 1 woman, aged between 22 years and 43 years, were assessed.

### Screening procedures

A short diagnostic screening was carried out as a screening for neurologic and/or psychiatric diseases. The symptomatology of the patient groups was determined using various inventories (e.g. Obsessive Compulsive Drinking Scale). In addition, affective aspects and personality factors were assessed using several questionnaires (e.g. Beck Depression Inventory (BDI), State-trait anxiety questionnaire (STAI)).

The BDI scores of patients (mean: 9.9) were significantly higher than those of healthy controls (mean: 3.2; p = 0.018). The STAI score did not differ significantly between patients (mean: 35.36) and the control group (mean: 29.79; p = 0.120).

### Picture material and stimulation paradigm

**Fig 1:**
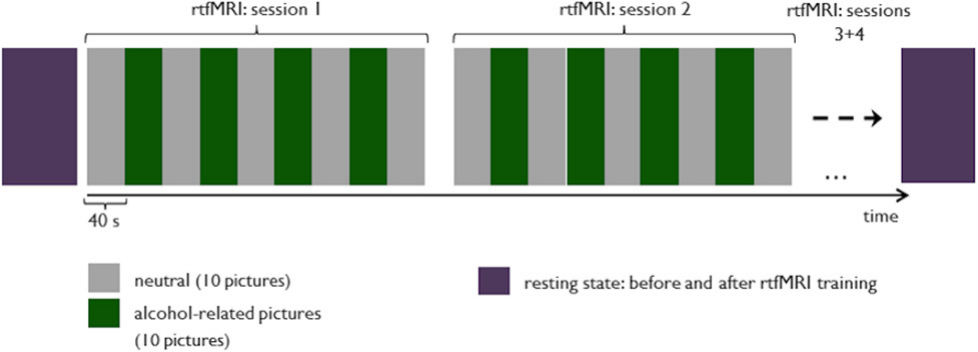
Experimental paradigm: presentation of neutral and alcohol-related pictures in blocks of 40 seconds with 10 pictures of the respective category; participants were instructed to reduce brain activity during the presentation of alcohol-associated information; during the presentation of neutral information, participants were instructed to simply gaze at the pictures [green: presentation of alcohol-related pictures; grey: presentation of neutral pictures].

The visual stimulation consisted of 30 alcohol-relevant pictures and 30 neutral pictures. Neutral pictures were taken from the International Affective Picture System (IAPS, http://csea.phhp.ufl.edu). The alcohol-relevant pictures showed specific triggers for alcohol consumption e.g. glasses of wine and/or beer, bottles of alcohol, persons drinking alcohol. All pictures were shown for four seconds, each presented in blocks of 40 seconds. Exactly the same picture sequence was presented to patients and their matched control subjects (see Fig 1).

FMRI measurements took place at the Institute for Clinical Radiology, Ludwig-Maximilians-University Munich. Patients and healthy subjects participated in an rtfMRI session. The fMRI session comprised two paradigms: (1) cue exposure and (2) neurofeedback paradigm.

*(1) Cue exposure*: Neutral pictures and addiction-related cues were presented to the participants. The pictures were presented in pseudo-randomised order. BOLD responses during the neutral condition and drug-related pictures were compared in order to define areas of the prefrontal cortex with increased BOLD signals in response to alcohol stimuli exposition which are linked specifically to cue exposures in each participant. An individual region of interest (ROI) was defined for each patient with *true* rtfMRI comprising the brain area with the most extensive addiction-related BOLD response in the anterior cingulate cortex (e.g. Childress et al. 1999), the dorsolateral prefrontal cortex (Georg et al. 2001) or the insula. In healthy subjects, the ROI was also placed in the prefrontal cortex comprising a brain area with enhanced BOLD responses during the presentation of alcohol-related information as compared to neutral pictures. In patients with *sham* rtfMRI, a brain area, which has demonstrated not to be influenced by the presentation of alcohol cues was selected for the ROI analysis (e.g. the cuneus).

*(2) Neurofeedback-paradigm (using Turbo-BrainVoyager*, *http*:*//www*.*brainvoyager*.*com/TurboBrainVoyager*.*html* *)* **:** During the rtfMRI sessions neutral and drug-related pictures were displayed in blocks of 40 seconds. The alcohol-related pictures were generated for the study and were not used in any other study before. During each block, each picture was presented for 4 seconds. During one single neurofeedback session, 5 blocks of neutral pictures and 4 blocks of addiction-related pictures were shown. Each block lasted about 6 minutes. Patients participated in 4 neurofeedback sessions taking place consecutively (see picture 1).

Patients with alcohol dependence disorder were divided into two subgroups:

*(1) True neurofeedback (experimental intervention)*: Neutral and alcohol-relevant pictures were presented together with the individual BOLD response in the individual *prefrontal cortex ROI*, which was identified during the cue exposure paradigm. The ROI activity was presented as continuous feedback using a “graphical thermometer” based on the top one-third of voxels with the highest t-values. Participants were instructed to decrease the responses of the feedback-thermometer during the presentation of addiction-cues. During the neutral condition, participants were asked to gaze at the pictures.

*(2) Sham neurofeedback (control condition)*: Apart from the pictures, individual BOLD responses of a sham ROI were presented to the participants as feedback via a thermometer. The sham ROI was placed in a brain area that is not related to craving and/or cue exposure (e.g. cuneus). Patients were instructed to decrease the responses of the feedback-thermometer during the presentation of addiction-cues.

### Resting state condition

A resting-state sequence (fcMRI) was included directly before and after the neurofeedback-training. The resting state-task took about 8 minutes before and after rtfMRI. For fcMRI data acquisition at rest, patients were instructed to keep their eyes closed without falling asleep, and try to think of nothing in particular. Measurements were acquired at exactly the same time of day to ensure equal inter-day testing conditions.

#### Assessment of the intensity of NF strategies and craving

After the fMRI scans, all participants attended a brief structured interview to determine the strategies which they employed to help them to reduce their craving. Craving was assessed before the resting state session outside the MRI scanner using the Obsessive Compulsive Drinking Scale [OCDS]. In addition, two questions about the current intensity of craving (urge to drink; control of drinking behaviour) were asked before and after the neurofeedback training. The results of these questions were summed up.

### MRI data acquisition and fMRI data analysis

FMRI-imaging was performed in a 3 Tesla Philips scanner with echo planar capability. A three-dimensional MPRAGE data set (T1-weighted) was acquired for each subject for anatomical referencing. For functional BOLD imaging during the neurofeedback-paradigm an EPI sequence was acquired in the same position as the anatomical images (repetition time: 2000 ms; echo time (TE): 35 ms; 25 axial slices; Field of View: 230 x 230 x 132 mm; spatial resolution: 3 x 3 x 3 mm; slice thickness: 4 mm; gap: 0.15 mm).

The initial processing and analysis as well as the feedback for the participants were done using the TurboBrainVoyager software package. The further post-processing of data and analysis of the fMRI data was carried out by the BrainVoyager software package (Brain Innovation, Maastricht, Netherlands). The first 5 images were excluded from any further analysis due to relaxation time effects. The preprocessing of the functional data included high-pass filtering (cutoff: three cycles in a time course) to low frequency signal drift inherent in echo planar imaging, a slice scan time correction, spatial smoothing (Gaussian filter with FWHM 8.0 mm), and a 3D motion correction. In addition, functional images were transferred to a standard Talairach brain. Significant BOLD activity was determined by a cross correlation of MR image pixel intensity with an expected hemodynamic response function. Voxelwise t-tests were used to identify those brain areas where the signal change was significantly different between alcohol-related responses and neutral stimuli. For each participant the conditions *alcohol-relevant pictures* and *neutral* were calculated as regressors.

In addition, the results of the individual ROIs were calculated: the percentage variations of the number of activated voxels during the presentation of alcohol relevant information minus neutral pictures were calculated separately for each participant before and after rtfMRI measurements.

### Resting-state paradigm: acquisition and analysis

Data for functional connectivity were acquired using an echo-planar imaging (EPI) sequence with the following parameters was used: Repetition time (TR): 2500 ms, echo time (TE): 25 ms, flip angle (FA): 90 deg., spatial resolution: 3 × 3 × 3 mm^3^, imaging matrix: 76 × 77, field-of-view (FoV): 230 × 230 × 132 mm, number of slices: 44, number of volumes: 180 and SENSE: 1.8 (p reduction, AP). The analysis of functional MRI connectivity was done as described previously in more detail (Keeser et al. 2011). The scripts have been updated to the latest version using the C-PAC code (http://fcon_1000.projects.nitrc.org/indi/cpac/index.html) and the software packages AFNI (http://afni.nimh.nih.gov/afni) and FSL, software version 5.0.6 were used. Preprocessed time series data from the entire data set (pre- and post-measurements) were concatenated across subjects to create a single 4D dataset that was decomposed using MELODIC (Multivariate Exploratory Linear Optimized Decomposition into Independent Components) built in FSL. Independent components were estimated using the automatic dimensionality estimation function in FSL. The dual regression approach was used to evaluate the Default Mode Network (DMN) pre- and post Neurofeedback training for visual purposes only as key regions (anterior cingulate cortex (ACC), BA 9) of the DMN were selected for the seed-based functional connectivity analysis. As head motion can influence fcMRI [48,49] the mean relative displacement (in mm) of each brain volume in relation to the previous one was estimated for the translation parameters xyz-direction across all time points.

### Seed-based analysis of functional connectivity

For the preprocessing of EPI (functional) and T1 (anatomical) data the Analysis of Functional Images (AFNI) software (Cox, 1996; http://afni.nimh.nih.gov/) was used. 180 Resting-State volumes were included in the analysis. The first three volumes of each scan were discarded to avoid equilibration effects using 3dTCat. For each volume the outlier fraction was computed using the tool 3dToutcount and data was „de-spiked”using 3dDespike. All slices were slice time corrected using 3dTshift. A 12-parameter affine transformation registered each participant’s anatomical scan with the TT_N27 template, and the anatomy was warped to Talairach space (Talairach & Tournoux, 1988). This transformation was also applied to the functional data and an 8 mm full-width half maximum (FWHM) Gaussian filter was applied in order to smooth the volumes accordingly. A six-parameter rigid body transformation corrected head motion was used, demeaned motion parameters and derivatives were calculated and excessive motion was censored. Subjects who exhibited excessive head motion were excluded from further analysis. A regression analysis was done including the average signal from white matter voxels, ventricle voxel activations, head motion and band-pass filtering (0.01–0.1) using AFNI’s 3dDeconvolve to remove nuisance signals from the voxel time course was adjusted. Subjects that exhibited excessive head motion were excluded from further analysis.

**Fig 2:**
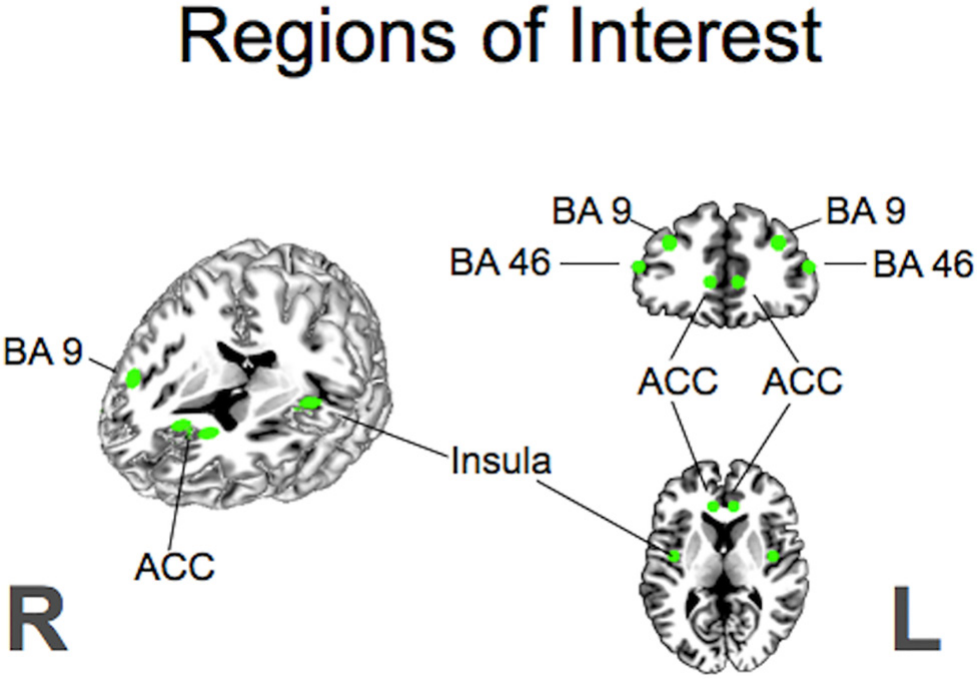
Regions defined for the seed-based functional connectivity analysis in areas are known to be relevant for addictive behaviour, including the left insula, the right insula, the left DLPFC (BA 9, 46) and the right DLPFC (BA 9, 46).

Binarised masks where built according to selected training regions and pre-known regions of interest in addictive behaviour: ACC, left insula, right insula, left DLPFC (BA 9, 46 separately), right DLPFC (BA 9, 46 separately), see Fig 2. The time course between these ROIs and all other voxels in the brain was calculated (seed-based functional connectivity analysis) using 3dmaskave and 3dfim+. In order to standardise outcome, z-score values were calculated using the formula:

$$\frac{\text{log}\frac{1+a}{1-a}}{2}$$

### Statistical Analysis

The OCDS ratings of patients and controls before NF were compared using t-tests for independent comparisons (Wilcoxon test).

For the assessment of subjective craving with two questions of the OCDS, an ANOVA was calculated with the within subject factor time (before NF, after NF) and the between subject factor group (patients *true* rtfMRI; controls *true* rtfMRI). Because of the variable sample sizes of the groups and the small sample size, especially of the patients sham rtfMRI group, their results were not integrated in a combined ANOVA. In addition, paired t-tests for the pre/-post-comparisons of craving before and after measurements were calculated for each group

Regarding the ROI analysis, an ANOVA was calculated with the within subject factor time (cue exposure paradigm and 4 NF sessions) and the between subject factor group (patients *true* rtfMRI; controls *true* rtfMRI). Because of the variable sample sizes of the groups and the small sample size, especially of the patients sham rtfMRI group, their results were not integrated in a combined ANOVA but separate ANOVAs for each group were calculated (patient *true* rtfMRI, patients *sham* rtfMRI, healthy subjects *true* rtfMRI, healthy subjects *sham* rtfMRI). The results were corrected for multiple comparisons (Bonferroni correction). All significance tests were two-sided. For all statistics SPSS 13.0 was used.

Using the dual regression approach to elucidate the DMN pre- and post to neurofeedback training a FWE-corrected threshold with p<0.05 was used. Paired t-tests (Wilcoxon test) were used to compare relative head motion post and pre to the neurofeedback training.

For the resting-state seed-based functional connectivity analysis a paired t-test was carried out for post vs. pre-real-time data using AFNI’s 3dtest ++ with a whole brain corrected α≤0.05 and a cluster extent ≥ 40 voxels. Seed regions: anterior cingulate cortex (R/L), insula (L/R), middle frontal area (BA 46; L/R) and dorsolateral prefrontal cortex (BA 9; L/R).

## Results

### True neurofeedback condition

#### Craving ratings

Subjective craving assessed by the German version of the Obsessive Compulsive Drinking Scale (OCDS) revealed enhanced scores in patients (before *true* rtfMRI: M = 11.73; SD = 6.842) compared to healthy controls (before *true* rtfMRI: M = 2.14; SD = 2.316; p < .001).

The ANOVA with the within subject factor time (before NF, after NF) and the between subject factor group (patients *true* rtfMRI, controls *true* rtfMRI) demonstrated a significant effect of time (F(1, 23) = 7.685; p = .011) for the assessment of the individual craving (questions about the urge to drink; control of drinking behaviour). Separate analyses for patients and controls showed that patients reached slightly decreased scores after rtfMRI (M = 1.82; SD = 1.722) compared to the ratings before rtfMRI training (M = 0.91; SD = 1.044). The differences reached trend level (p = .083).

#### NF fMRI results

**Fig 3:**
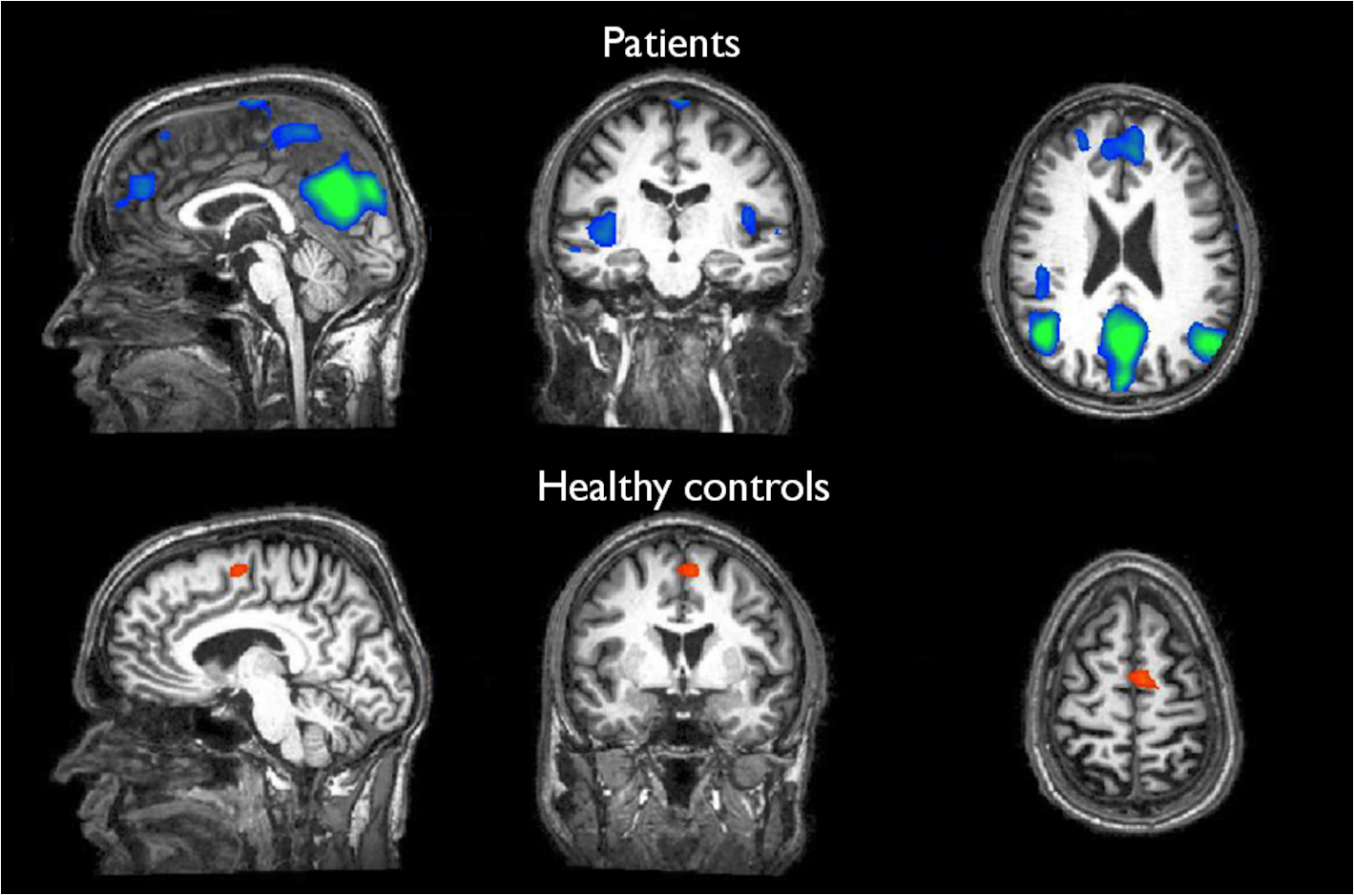
Comparison of neuronal responses during the fourth neurofeedback-session compared to the first neurofeedback-session (alcohol-related pictures > neutral pictures) in patients as well as healthy controls with *true* rtfMRI: Patients: reduced activity especially in the insula, the ACC, the medial frontal gyrus, the inferior temporal gyrus and the cuneus (p(Bonf)<0.0001; x = 0; y = -16; z = 22). Healthy controls: slightly increased activity especially in the medial frontal gyrus (p(Bonf)<0.0001; x = -7; y = -1; z = 60).

**Fig 4:**
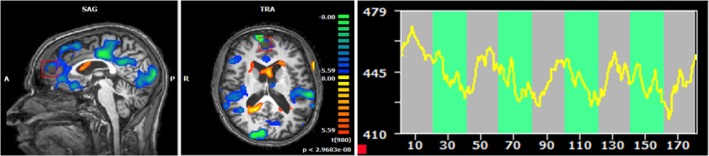
Results of a single patient with true neurofeedback: A) Neuronal response in the ROI during the fourth neurofeedback session compared to the first session [alcohol-related pictures > neutral pictures]. B) Time course of activity within the specific ROI during the fourth neurofeedback session [green: presentation of alcohol-related pictures; grey: presentation of neutral pictures; p(Bonf)<0.002; x = 3; y = 57; z = 15].

Patients from the *true* rtfMRI group showed reduced BOLD responses during the presentation of alcohol-relevant information in the fourth neurofeedback session compared to the neuronal activity in the cue exposure task, especially in the ACC, the insula, the dorsolateral prefrontal cortex, the medial frontal gyrus, the inferior temporal gyrus, the cuneus and parietal areas (see Fig 3; Table 1). However, the variability of the results between patients was great, sometimes showing a strong correspondence between neuronal response and task during the fourth neurofeedback session (see Fig 4); in other cases, the association was not clearly found.

**Table 1:** Functional variations of patients and controls. Neuronal responses during the 4th neurofeedback session compared to the 1st neurofeedback session (alcohol-related pictures minus neutral pictures) in patients and healthy controls (p(Bonf) < 0.0001; T-score: 6.7–10.2; abbreviations: BA: Brodman area; L: left; R: right; t-max: maximal t-score; Ø t-score: average t-score; voxels: number of activated voxels; x: Talairach coordinate x-axis; y: Talairach coordinate y-axis; z: Talairach coordinate z-axis)

Patients								
brain region	BA	L/R	Ø t-score	t-max	voxels	centre of gravity		
						x	y	z
*frontal lobe*								
inferior frontal gyrus	47	R	-8.04	-11.21	2220	45	31	-7
medial frontal gyrus	9	R	-7.50	-10.40	11664	4	49	26
superior frontal gyrus	6	R	-7.37	-10.17	32995	11	28	57
	8	L	-7.95	-10.02	1246	-10	38	53
	8	L	-7.45	-8.95	2232	-29	21	52
insula	13	R	-7.24	-8.54	4315	44	-19	1
*temporal lobe*								
superior temporal gyrus	39	L	-8.27	-10.61	7284	-49	-61	19
	22	L	-7.01	-8.31	2042	-48	-20	4
medial temporal gyrus	39	R	-8.55	-12.02	7841	45	-57	22
*subcortical*								
ncl. lenitformis/putamen		R	-7.21	-8.29	937	23	10	-12
*diverse*								
paracentral gyrus	5	L	-7.25	-8.86	6189	-2	-38	58
postcentral gyrus	4	R	-7.49	-9.26	2103	29	-30	55
precuneus	23	L	-8.52	-13.48	26231	0	-64	21
**Healthy controls**								
medial frontal gyrus	6	L	7.07	7.64	626	-4	-3	58

By contrast, in healthy subjects BOLD responses during the presentation of alcohol cues did hardly change between the first cue-exposure session compared to the fourth neurofeedback trial: neuronal responses were slightly increased in the medial prefrontal cortex; all other brain areas did not show any variations (see Fig 3; Table 1).

#### Results of the ROI analysis

The ANOVA, including the results of the *true* rtfMRI groups (patients and controls), demonstrated a significant effect of NF: the percentage number of activated voxels decreased during the NF training (F(4, 92) = 2,621; p = 0,040). The interaction of group and time (F(4, 92) = 1,435; p = 0,229) and the between subject effect were not significant (F(1, 23) = 0,042; p = 0,840).

A separate ANOVA for patients during true fMRI revealed a significant differences regarding the percentage of activated voxels during the presentation of alcohol-related information in the individually defined ROI training across sessions: The percentage of activated voxels was higher before the neurofeedback training compared to subsequent neurofeedback-sessions (F(4, 40) = 2.631; p = .048). A significant reduction was demonstrated in the comparison of responses during the cue-exposure before rtfMRI compared to the third neurofeedback trial (p = .023); the difference between cue exposure and the first rtfMRI trials reached trend level (p = .059). However, the results within the true rtfMRI group varied considerably: in some patients the BOLD response was manipulated considerably whereas other patients were not able to regulate their BOLD responses significantly.

In healthy subjects, BOLD responses did not decrease significantly during the neurofeedback-training (F(4, 52) = 1.338; p = .268).

### Sham neurofeedback condition

#### Craving ratings

Craving ratings of patients (p = .317) and controls (p = .180) of the sham NF-group was not influenced by neurofeedback-training.

#### ROI-analysis

The percentage of activated voxels during the presentation of alcohol-related pictures did not change significantly during neurofeedback in patients (F(4, 4) = 1.767; p = .298) and healthy controls (F(4, 40) = 1.801; p = 0.542).

### Results of the head motion comparison

The post vs. pre comparisons of the fcMRI time series data in regard to head motions by the mean relative displacements (in mm) revealed no significant differences (mean relative displacement prior to neurofeedback: 0.1175±0.0586, post to neurofeedback: 0.0957±0.0659, p = 0.208).

### Results of the resting state paradigm

#### Default mode network

**Fig 5:**
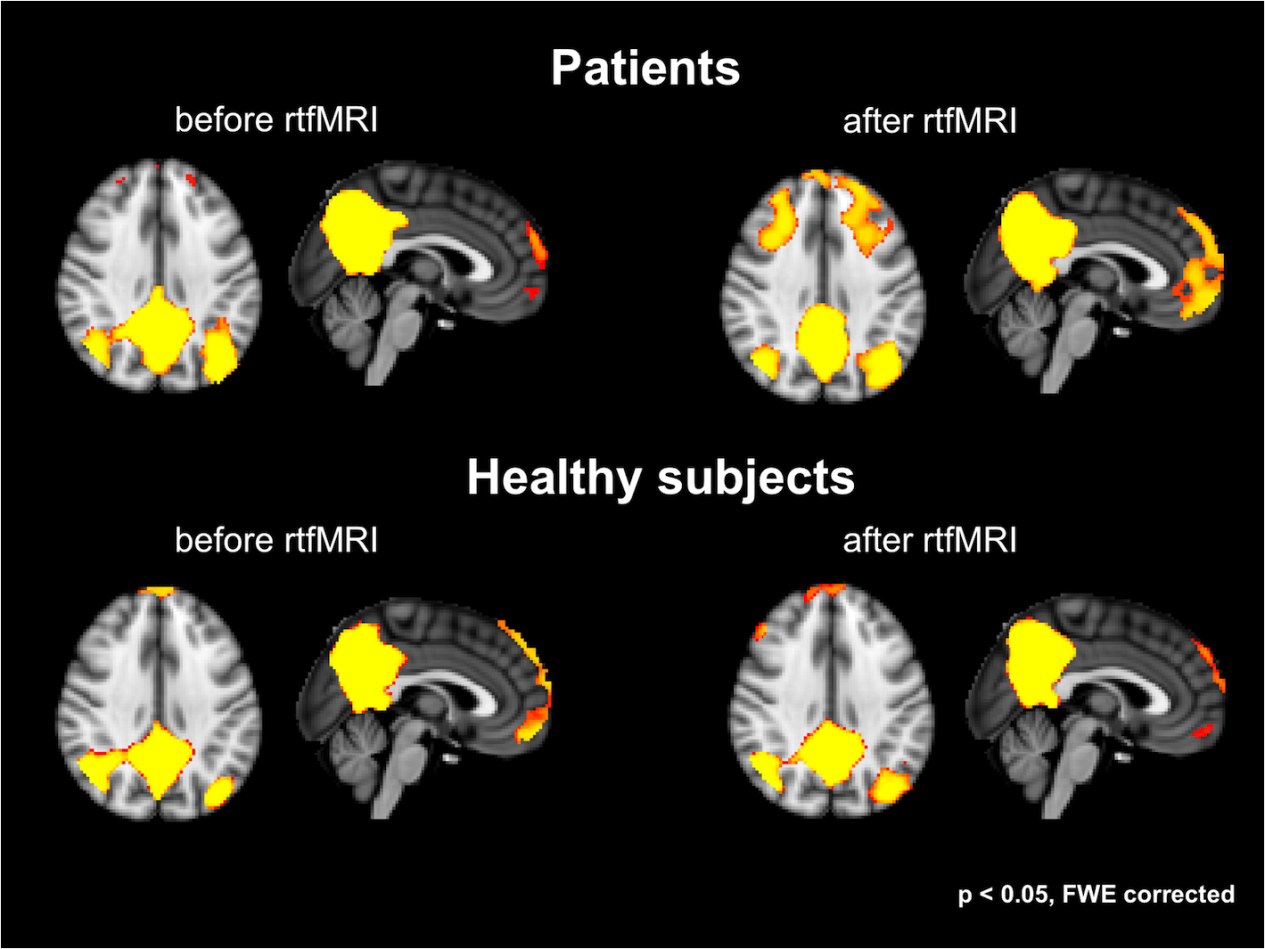
Results of the default mode network in patients and controls: increased medial frontal and dorsolateral prefrontal responses after rtfMRI in patients; in healthy controls, the activity was comparable before and after rtfMRI (p < 0.05, FWE corrected).

Patients demonstrated enhanced activations, especially in medial frontal areas as well as the dorsolateral-prefrontal cortex after *true* rtfMRI sessions. In healthy controls differences regarding the DMN activity before and after neurofeedback training did slightly change (see Fig 5).

#### Seed-based results

**Fig 6:**
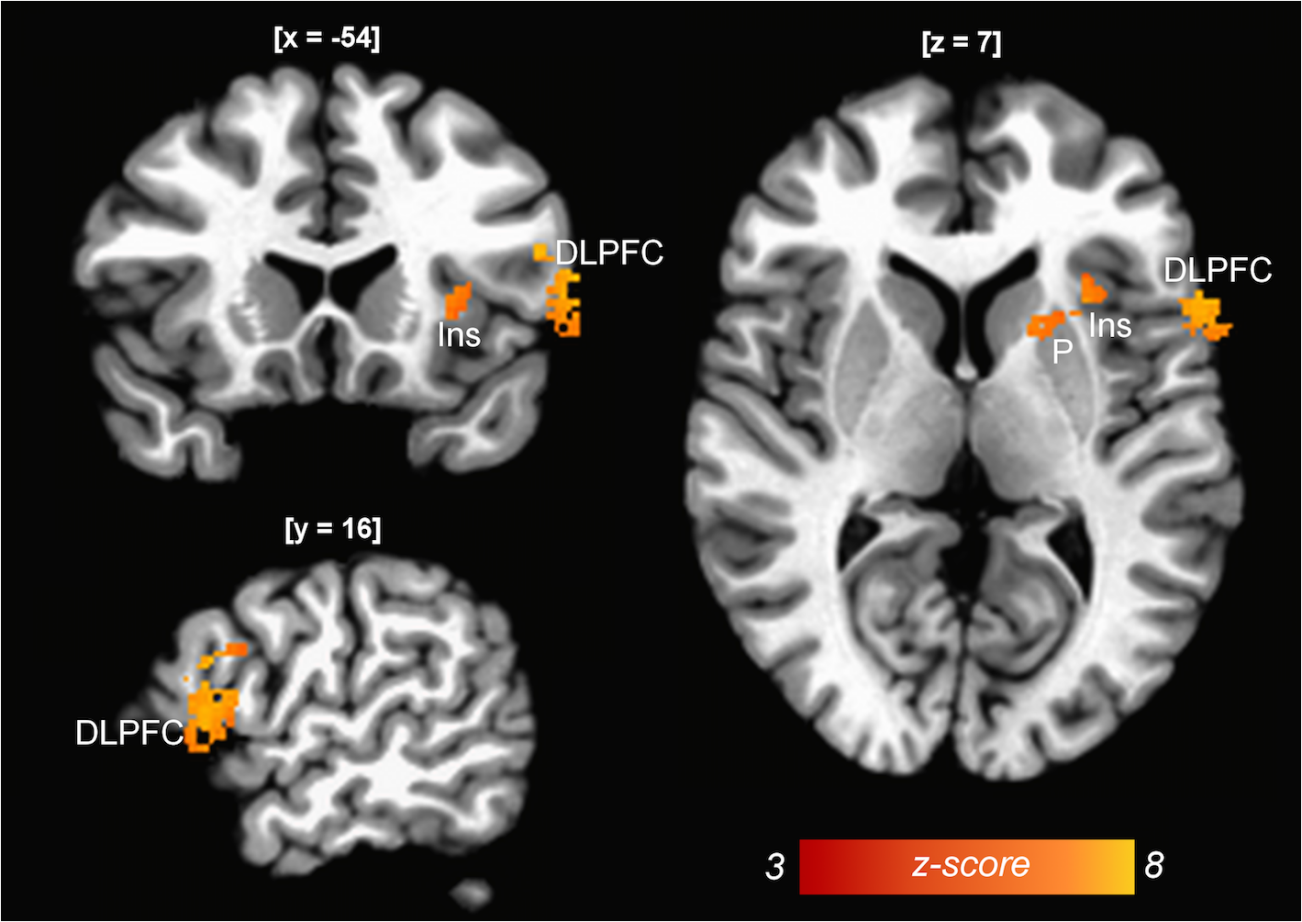
Seed based fMRI analysis: Comparison of resting state functional connectivity before and after neurofeedback training (p(FEW) < 0.05). After neurofeedback, increased functional connectivity was detected between the left Brodman Area 46 and different cortical and subcortical brain regions, including the dorsolateral prefrontal cortex (DLPFC), the insula (Ins) and the putamen (P) of the ipsilateral hemisphere. Connectivity patterns are superimposed on the TT N27-template. Images are displayed in the radiological convention (coordinates in Talairach space are given in parenthesis).

Patients demonstrated increased connections after the rtfMRI compared to before the neurofeedback-training between the left ACC and the thalamus, temporal areas as well as the dorsal part of the ACC. Increased connections were also seen between the left insula and the medial prefrontal gyrus/superior frontal gyrus as well as parietal areas, between insula right and the orbitofrontal cortex/medial frontal gyrus and temporal regions. The most prominent increases occurred between the left middle frontal area (BA 46) and the dorsolateral prefrontal cortex, lentiform nucleus, thalamus/claustrum and the parahippocampal gyrus; the connection between the right middle frontal area and the insula seemed to be increased as well (see Fig 6, Table 2).

**Table 2:** Changes in connectivity in patients and controls. Variations in the connectivity in patients and healthy subjects; paired t-tests were done for post-real-time fMRI data compared to pre-real-time fMRI data with an entire brain analysis corrected with ∝ ≤ .05 and a cluster level of ≥ 40 voxels; Talairach coordinates; coordinates are displayed in LPI format (abbreviations: L: left; R: right; voxels: number of voxels; mid.: middle; ↑: increase; ↓: decrease).

Patients						
Seed region	Target region	Voxels	x	y	z	↑/↓
BA 9 R	medial frontal gyrus/subcallosal gyrus	67	3	21	-13	↓
BA 9 L	-	-	-	-	-	-
BA 46 R	Insula	59	-48	9	4	↑
	inferior parietal lobule	44	63	-25	25	↑
BA 46 L	inferior frontal gyrus (BA 44)	388	-51	13	17	↑
	lentiform gyrus/putamen	216	-22	3	17	↑
	claustrum/pulvinar	195	-24	-22	16	↑
	insula/claustrum	69	35	-15	-2	↑
		44	-28	15	7	↑
ACC R	postcentral/paracentral lobule (BA 40)	44	-24	44	54	↑
	posterior cingulate (BA 30)	75	2	60	10	↓
	Cerebellum	110	-30	72	-33	↓
ACC L	thalamus/pulvinar	146	-6	-24	6	↓
	uncus/parahippocampal gyrus (BA 36)	111	-21	-1	-26	↓
	Cerebellum	54	29	-42	-37	↓
		48	29	-54	-26	↓
	pre-/postcentral gyrus	71	-46	—7	25	↑
	parahippocampal gyrus	63	27	-28	-17	↑
insula R	superior frontal /middle temporal gyrus	59	30	3	-35	↓
insula L	medial frontal gyrus (BA 6)	49	-12	3	53	↓
**Healthy**	**Controls**					
BA 9 R	-	-	-	-	-	
BA 9 L	ACC/mid. frontal gyrus (BA 24)	175	5	11	34	↓
	superior frontal gyrus (BA 6)	145	-15	3	52	↓
		50	18	23	52	↓
	middle/superior frontal gyrus (BA 6)	40	27	9	55	↓
	lingual gyrus/parahippocampal gyrus (BA 19)	98	-18	-42	-4	↓
	posterior cingulate	69	-9	-45	25	↓
BA 46 R	-	-	-	-	-	
BA 46 L	ACC (BA 9)	114	12	40	16	↑
ACC R	middle cingulate cortex	169	-7	-21	43	↓
	left cerebellum	49	-30	-69	-44	↑
	insula/claustrum	43	-34	-4	7	↓
ACC L	precentral gyrus (BA 6)	344	57	0	31	↓
	postcentral gyrus (BA 4)	113	-52	-15	23	↓
	middle/superior temporal lobe (BA 41/22)	105	-51	-25	4	↓
	precentral lobe/cingulate cortex	93	-9	-19	43	↓
	superior frontal gyrus/SMA	79	-13	5	47	↓
	left cerebellum	50	-19	-46	-52	↓
	superior orb. gyrus (BA 10)	50	-28	59	5	↓
	middle cingulate cortex	42	11	-19	41	↓
insula R	fusiform gyrus	111	-39	-64	-13	↑
	precuneus	72	8	-58	29	↑
	medial frontal gyrus (BA 10)	52	-12	65	5	↓
insula L	putamen/ncl. lentiformis	75	-25	10	8	↓
	superior frontal gyrus	46	-18	-1	44	↓

Connections between the right ACC, the left and right dorsolateral prefrontal cortex (BA 9) did not change in consequence of the rtfMRI paradigm.

## Discussion and Conclusion

The aim of the present project was to investigate the feasibility of rtfMRI neurofeedback in order to influence both neuronal responses and craving to addiction-related cues in patients with alcohol use disorder. In *patients with alcohol use disorder* we found a significant reduction of neuronal activity in the brain regions used as a target area for the neurofeedback-training (e.g. ACC, DLPFC, insula) during cue-induced craving. Effective modulation of cue-elicited craving is crucial to successful substance withdrawal. Neurofeedback-related changes seemed to be specific for patients; ROI-based functional responses did not change significantly in *healthy controls*.

Corresponding results were demonstrated in the DMN: in patients, the responses in the medial frontal areas as well as the dorsolateral prefrontal cortex were enhanced after the neurofeedback-training compared to baseline. In addition, the seed-based functional *connectivity between brain areas* changed after neurofeedback significantly: patients with alcohol-use disorder revealed increased connections between the middle frontal area (BA 46) and other frontal regions (e.g. dorsolateral prefrontal cortex, insula) as well as subcortical areas (e.g. lentiform nucleus, thalamus/claustrum, parahippocampal gyrus). Enhanced connections were also seen between various frontal regions e.g. between the insula and medial/superior frontal gyrus as well as the orbitofrontal gyrus/medial frontal cortex. In addition, functional connections between frontal parts of the brain and temporal and parietal areas demonstrated to be more pronounced. It is plausible that the changes in the BOLD-signal of specific regions modulate the connectivity not only in this region but also in more distant regions highlighting the interconnectivity of the human brain. DMN was only slightly modulated by the neurofeedback-training in the control group.

Altogether, these results may provide some evidence for functional variations especially within the frontal cortex as well as between frontal parts of the brain and other areas, which have been shown to be specifically relevant in substance use disorders (e.g. subcortical areas). These effects are not restricted to the neurofeedback-training session but seemed to be present also during resting state condition.

The importance of frontal brain responses to neurofeedback-training in patients with substance use disorders has been shown before. One former study on neurofeedback in patients with nicotine dependent smokers has demonstrated that treatment-seeking smokers were able to decrease brain responses in craving-relevant areas like the ACC [11,46]. In addition, activity of the ventral ACC did correlate with craving ratings. The authors concluded that ACC activation and craving could be modulated by neurofeedback. Canterberry and colleagues (2013) also demonstrated decreased activity across several visits in patients with nicotine dependence in the ACC after a neurofeedback-training. Changes in ACC activity were specific to disorder-related information and did not emerge during the presentation of neutral stimuli [45]. The neuronal responses were influenced by the severity of nicotine dependence: Neurofeedback tended to be more effective in people with lower nicotine dependence. These results are consistent with those of other studies showing that the level of dependence affects treatment outcome [50].

Several of the areas affected during our study are thought to be part of the so-called reward system which is specifically important for motivation, evaluation of actions as well as the mediation of the effects of reinforcement/reduction of behaviour. The reward system plays a major role for adaptive behaviour, control of behaviours and learning processes. Neuroimaging studies have demonstrated that various brain areas are included e.g. the ACC and adjacent medial frontal areas, orbitofrontal cortex, amygdala, hypothalamus, dorsal/ventral striatum, ncl. accumbens. The importance of the reward system in substance use disorder has been demonstrated in many studies.

The functional results of patients were associated with a slightly reduced craving after real-time fMRI training compared to the assessment of craving before the training. This may indicate that the modulation of brain activity leads to changes of the evaluation, behavioural responses and/or affective responsiveness to disorder-relevant information. The assessment of other clinical parameters was only done before the neurofeedback training. For that reason, we cannot draw any conclusions about the effect of neurofeedback on depression or anxiety.

Up to now, only few studies addressed the influence of ROI-based neurofeedback on brain connectivity. Ruiz and colleagues (2013) were able to demonstrate that learned regulation of the activity in the insular cortex leads to an enhanced effective connectivity of the emotional network (e.g. connections among insula cortex, amygdala and the medial prefrontal cortex) in schizophrenic patients. The authors assumed that this indicates that schizophrenic patients are capable of a volitional regulation of brain responses using rtfMRI after sufficient training. The learned self-regulation led to changes in the perception of emotional faces [47]. The insular cortex is a key region of salience processes including sensory and cognitive processing (39).

In the present study, reduced insular activation in alcohol patients may have directly contributed to the changes in craving found after the neurofeedback-training. The increased connectivity comprised not only the insula but also the left DLPFC. The increased functional connectivity in the DLPFC may support cognitive control and encounters cravings whereas the increased insular connectivity may decrease the emotional content of nicotine-associated cues. These findings can contribute to another hypothesis generation and have to be further elucidated in future studies.

One main aim of real-time neurofeedback is that the effect of neurofeedback training should be persistent and patients should be able to generalise cognitive strategies which were generated during neurofeedback can be used in the natural environment. The present study already demonstrated ongoing effects of the neurofeedback training during the resting-state condition directly after the neurofeedback sessions. However, further assessments of neuronal responses after longer intervals are needed in order to evaluate the persistence of these responses. Whether there is a stable therapeutic effect of reduced craving and to which extent this might be the case needs to be investigated in further studies.

Altogether, the results of the present study demonstrate that it is feasible for patients with alcohol dependency to reduce their neuronal activity using rtfMRI. In addition, there is some evidence that craving can be influenced by this technique. However, there were also comparatively big within-group differences regarding patient group. Possible adaptions of the training program could be useful: the length of the neurofeedback sessions should maybe be shortened in order to increase the motivation during the session. In addition, more specific training strategies might be beneficial for the patients to be able to manage the regulation of craving-related brain responses more easily. Apart from these strategies, repeated neurofeedback sessions could probably lead to a higher proportion of patients who can reduce their neuronal activity in the ROI, make the effects more stable and enduring, and might decrease craving more effectively. Hanlon and colleagues (2013), for example, have demonstrated that the benefit of neurofeedback was maximised during the third visit. In addition, with the aid of more neurofeedback sessions a more specific reduction of activity only in the ROI may be learned.

Moreover, control groups should be included to increase specificity (e.g. due to habituation). The application of neurofeedback training to patients with other addiction disorders (e.g. pathological gambling, tobacco dependence) or different psychiatric disorders (e.g. affective disorders) would help to evaluate the generalisability of this approach

We are aware of the fact that age does alter brain function and it is likely that neuroplasticity decreases with age. However, our study was conceptualised as an initial pilot study in an exploratory manner. Further studies and an increased sample size are needed to confirm or reject our findings when incorporating patients with a different age range.

Overall the results of the current study support the assumption that real-time fMRI may be a valid therapeutic tool for patients with substance use disorder. However, further studies are needed to improve these strategies and to examine possible influencing parameters in order to reduce the high variability between subjects.

## References

[1] World_Health_Organization. International Statistical Classification of Diseases and Related Health Problems. (2010). Geneva: World Health Organization.

[2] Everitt BJ, Robbins TW. Neural systems of reinforcement for drug addiction: from actions to habits to compulsion. Nat Neurosci. 2005; 8: 1481–1489. 16251991 10.1038/nn1579

[3] Di Chiara G, Bassareo V. Reward system and addiction: what dopamine does and doesn't do. Curr Opin Pharmacol. 2007; 7: 69–76. 17174602 10.1016/j.coph.2006.11.003

[4] Heinz A, Beck A, Grusser SM, Grace AA, Wrase J. Identifying the neural circuitry of alcohol craving and relapse vulnerability. Addict Biol. 2009; 14: 108–118. 10.1111/j.1369-1600.2008.00136.x 18855799 PMC2879014

[5] Heinz A, Siessmeier T, Wrase J, Hermann D, Klein S, Grusser SM, et al Correlation between dopamine D(2) receptors in the ventral striatum and central processing of alcohol cues and craving. The American journal of psychiatry. 2004; 161: 1783–1789. 15465974 10.1176/appi.ajp.161.10.1783

[6] Wrase J, Grusser SM, Klein S, Diener C, Hermann D, Flor H, et al Development of alcohol-associated cues and cue-induced brain activation in alcoholics. Eur Psychiatry. 2002; 17: 287–291. 12381499 10.1016/s0924-9338(02)00676-4

[7] Myrick H, Li X, Randall PK, Henderson S, Voronin K, Anton RF. The effect of aripiprazole on cue-induced brain activation and drinking parameters in alcoholics. J Clin Psychopharmacol. 2004; 30: 365–372. 10.1097/JCP.0b013e3181e75cff PMC3595185 20571434

[8] George MS, Anton RF, Bloomer C, Teneback C, Drobes DJ, Lorberbaum JP, et al Activation of prefrontal cortex and anterior thalamus in alcoholic subjects on exposure to alcohol-specific cues. Arch Gen Psychiatry. 2001; 58: 345–352. 11296095 10.1001/archpsyc.58.4.345

[9] Wrase J, Schlagenhauf F, Kienast T, Wustenberg T, Bermpohl F, Kahnt T, et al Dysfunction of reward processing correlates with alcohol craving in detoxified alcoholics. Neuroimage. 2007; 35: 787–794. 17291784 10.1016/j.neuroimage.2006.11.043

[10] Grusser SM, Wrase J, Klein S, Hermann D, Smolka MN, Ruf M, et al Cue-induced activation of the striatum and medial prefrontal cortex is associated with subsequent relapse in abstinent alcoholics. Psychopharmacology (Berl). 2004; 175: 296–302. 15127179 10.1007/s00213-004-1828-4

[11] Li X, Hartwell KJ, Borckardt J, Prisciandaro JJ, Saladin ME, Morgan PS, et al Volitional reduction of anterior cingulate cortex activity produces decreased cue craving in smoking cessation: a preliminary real-time fMRI study. Addict Biol. 2013; 18: 739–748. 10.1111/j.1369-1600.2012.00449.x 22458676 PMC3389595

[12] Caria A, Sitaram R, Birbaumer N. Real-time fMRI: a tool for local brain regulation. Neuroscientist. 2012; 18: 487–501. 21652587 10.1177/1073858411407205

[13] de Ruiter MA, Meeteren AY, van Mourik R, Janssen TW, Greidanus JE, Oosterlaan J, et al Neurofeedback to improve neurocognitive functioning of children treated for a brain tumor: design of a randomized controlled double-blind trial. BMC Cancer. 2012; 12: 581 10.1186/1471-2407-12-581 23217162 PMC3530427

[14] Nazari MA, Mosanezhad E, Hashemi T, Jahan A. The effectiveness of neurofeedback training on EEG coherence and neuropsychological functions in children with reading disability. Clin EEG Neurosci. 2012; 43: 315–322. 10.1177/1550059412451880 23185091

[15] Schmidt T, Henrich D. Patient Adaptive Neurofeedback for ADHD Therapy. Biomed Tech (Berl). 2012.

[16] Arnold LE, Lofthouse N, Hersch S, Pan X, Hurt E, Bates B, et al EEG Neurofeedback for ADHD: Double-Blind Sham-Controlled Randomized Pilot Feasibility Trial. J Atten Disord. 2012. 10.1177/1087054712446173 PMC3939717 22617866

[17] Gevensleben H, Rothenberger A, Moll GH, Heinrich H. Neurofeedback in children with ADHD: validation and challenges. Expert Rev Neurother. 2012; 12: 447–460. 10.1586/ern.12.22 22449216

[18] Birbaumer N, Ghanayim N, Hinterberger T, Iversen I, Kotchoubey B, Kubler A, et al A spelling device for the paralysed. Nature. 1999; 398: 297–298. 10192330 10.1038/18581

[19] Kaiser J, Perelmouter J, Iversen IH, Neumann N, Ghanayim N, Hinterberger T, et al Self-initiation of EEG-based communication in paralyzed patients. Clin Neurophysiol. 2001; 112: 551–554. 11222979 10.1016/s1388-2457(01)00470-9

[20] Kubler A, Neumann N, Kaiser J, Kotchoubey B, Hinterberger T, Birbaumer NP. Brain-computer communication: self-regulation of slow cortical potentials for verbal communication. Arch Phys Med Rehabil. 2001; 82: 1533–1539. 11689972 10.1053/apmr.2001.26621

[21] Kubler A, Kotchoubey B, Kaiser J, Wolpaw JR, Birbaumer N. Brain-computer communication: unlocking the locked in. Psychol Bull. 2001; 127: 358–375. 11393301 10.1037/0033-2909.127.3.358

[22] Kotchoubey B, Strehl U, Uhlmann C, Holzapfel S, Konig M, Froscher W, et al Modification of slow cortical potentials in patients with refractory epilepsy: a controlled outcome study. Epilepsia. 2001; 42: 406–416. 11442161 10.1046/j.1528-1157.2001.22200.x

[23] Weiskopf N, Sitaram R, Josephs O, Veit R, Scharnowski F, Goebel R, et al Real-time functional magnetic resonance imaging: methods and applications. Magn Reson Imaging. 2007; 25: 989–1003. 17451904 10.1016/j.mri.2007.02.007

[24] Sitaram R, Caria A, Veit R, Gaber T, Rota G, Kuebler A, et al FMRI brain-computer interface: a tool for neuroscientific research and treatment. Comput Intell Neurosci. 2007; 25487 10.1155/2007/25487 18274615 PMC2233807

[25] Lee S, Ruiz S, Caria A, Veit R, Birbaumer N, Sitaram R. Detection of cerebral reorganization induced by real-time fMRI feedback training of insula activation: a multivariate investigation. Neurorehabil Neural Repair. 2011; 25: 259–267. 10.1177/1545968310385128 21357528

[26] deCharms RC. Reading and controlling human brain activation using real-time functional magnetic resonance imaging. Trends Cogn Sci. 2007; 11: 473–481. 17988931 10.1016/j.tics.2007.08.014

[27] Yoo SS, Jolesz FA. Functional MRI for neurofeedback: feasibility study on a hand motor task. Neuroreport. 2002; 13: 1377–1381. 12167756 10.1097/00001756-200208070-00005

[28] Posse S, Binkofski F, Schneider F, Gembris D, Frings W, Habel U, et al A new approach to measure single-event related brain activity using real-time fMRI: feasibility of sensory, motor, and higher cognitive tasks. Hum Brain Mapp. 2001; 12: 25–41. 11198103 10.1002/1097-0193(200101)12:1<25::AID-HBM30>3.0.CO;2-H PMC6871962

[29] deCharms RC, Christoff K, Glover GH, Pauly JM, Whitfield S, Gabrieli JD. Learned regulation of spatially localized brain activation using real-time fMRI. Neuroimage. 2004; 21: 436–443. 14741680 10.1016/j.neuroimage.2003.08.041

[30] Weiskopf N, Mathiak K, Bock SW, Scharnowski F, Veit R, Grodd W, et al Principles of a brain-computer interface (BCI) based on real-time functional magnetic resonance imaging (fMRI). IEEE Trans Biomed Eng. 2004; 51: 966–970. 15188865 10.1109/TBME.2004.827063

[31] Caria A, Veit R, Sitaram R, Lotze M, Weiskopf N, Grodd W, et al Regulation of anterior insular cortex activity using real-time fMRI. Neuroimage. 2007; 35: 1238–1246. 17336094 10.1016/j.neuroimage.2007.01.018

[32] Dyck M, Loughead J, Kellermann T, Boers F, Gur RC, Mathiak K. Cognitive versus automatic mechanisms of mood induction differentially activate left and right amygdala. Neuroimage. 2011; 54: 2503–2513. 10.1016/j.neuroimage.2010.10.013 20946960

[33] Posse S, Fitzgerald D, Gao K, Habel U, Rosenberg D, Moore GJ, et al Real-time fMRI of temporolimbic regions detects amygdala activation during single-trial self-induced sadness. Neuroimage. 2003; 18: 760–768. 12667853 10.1016/s1053-8119(03)00004-1

[34] Johnston SJ, Boehm SG, Healy D, Goebel R, Linden DE. Neurofeedback: A promising tool for the self-regulation of emotion networks. Neuroimage. 2010; 49: 1066–1072. 10.1016/j.neuroimage.2009.07.056 19646532

[35] Hamilton JP, Glover GH, Hsu JJ, Johnson RF, Gotlib IH. Modulation of subgenual anterior cingulate cortex activity with real-time neurofeedback. Hum Brain Mapp. 2011; 32: 22–31. 10.1002/hbm.20997 21157877 PMC3049174

[36] McCaig RG, Dixon M, Keramatian K, Liu I, Christoff K. Improved modulation of rostrolateral prefrontal cortex using real-time fMRI training and meta-cognitive awareness. Neuroimage. 2011; 55: 1298–1305. 10.1016/j.neuroimage.2010.12.016 21147230

[37] Johnston S, Linden DE, Healy D, Goebel R, Habes I, Boehm SG. Upregulation of emotion areas through neurofeedback with a focus on positive mood. Cogn Affect Behav Neurosci. 2011; 11: 44–51. 10.3758/s13415-010-0010-1 21264651

[38] Zotev V, Krueger F, Phillips R, Alvarez RP, Simmons WK, Bellgowan P, et al Self-regulation of amygdala activation using real-time FMRI neurofeedback. PLoS One. 2011; 6: e24522 10.1371/journal.pone.0024522 21931738 PMC3169601

[39] Caria A, Sitaram R, Veit R, Begliomini C, Birbaumer N. Volitional control of anterior insula activity modulates the response to aversive stimuli. A real-time functional magnetic resonance imaging study. Biological psychiatry. 2010; 68: 425–432. 10.1016/j.biopsych.2010.04.020 20570245

[40] deCharms RC, Maeda F, Glover GH, Ludlow D, Pauly JM, Soneji D, et al Control over brain activation and pain learned by using real-time functional MRI. Proc Natl Acad Sci U S A. 2005; 102: 18626–18631. 16352728 10.1073/pnas.0505210102 PMC1311906

[41] Rota G, Sitaram R, Veit R, Erb M, Weiskopf N, Dogil G, et al Self-regulation of regional cortical activity using real-time fMRI: the right inferior frontal gyrus and linguistic processing. Hum Brain Mapp. 2009; 30: 1605–1614. 10.1002/hbm.20621 18661503 PMC6870622

[42] Scheinost D, Stoica T, Saksa J, Papademetris X, Constable RT, Pittenger C, et al Orbitofrontal cortex neurofeedback produces lasting changes in contamination anxiety and resting-state connectivity. Transl Psychiatry. 2013; 3: e250 10.1038/tp.2013.24 23632454 PMC3641411

[43] Chapin H, Bagarinao E, Mackey S. Real-time fMRI applied to pain management. Neurosci Lett. 2012; 520: 174–181. 10.1016/j.neulet.2012.02.076 22414861 PMC3377818

[44] Linden DE, Habes I, Johnston SJ, Linden S, Tatineni R, Subramanian L, et al Real-time self-regulation of emotion networks in patients with depression. PLoS One. 2012; 7: e38115 10.1371/journal.pone.0038115 22675513 PMC3366978

[45] Canterberry M, Hanlon CA, Hartwell KJ, Li X, Owens M, LeMatty T, et al Sustained reduction of nicotine craving with real-time neurofeedback: exploring the role of severity of dependence. Nicotine Tob Res. 2013; 15: 2120–2124. 10.1093/ntr/ntt122 23935182 PMC3819983

[46] Hanlon CA, Hartwell KJ, Canterberry M, Li X, Owens M, Lematty T, et al Reduction of cue-induced craving through realtime neurofeedback in nicotine users: the role of region of interest selection and multiple visits. Psychiatry Res. 2013; 213: 79–81. 10.1016/j.pscychresns.2013.03.003 23683344 PMC4093788

[47] Ruiz S, Lee S, Soekadar SR, Caria A, Veit R, Kircher T, et al Acquired self-control of insula cortex modulates emotion recognition and brain network connectivity in schizophrenia. Hum Brain Mapp. 2013; 34: 200–212. 10.1002/hbm.21427 22021045 PMC6869886

[48] Power JD, Barnes KA, Snyder AZ, Schlaggar BL, Petersen SE. Spurious but systematic correlations in functional connectivity MRI networks arise from subject motion. Neuroimage. 2012; 59: 2142–2154. 10.1016/j.neuroimage.2011.10.018 22019881 PMC3254728

[49] Van Dijk KR, Sabuncu MR, Buckner RL. The influence of head motion on intrinsic functional connectivity MRI. Neuroimage. 2012; 59: 431–438. 10.1016/j.neuroimage.2011.07.044 21810475 PMC3683830

[50] Fagerstrom K, Furberg H. A comparison of the Fagerstrom Test for Nicotine Dependence and smoking prevalence across countries. Addiction. 2008; 103: 841–845. 10.1111/j.1360-0443.2008.02190.x 18412764 PMC2914535

